# Tobacco and E-Cigarettes Point of Sale Advertising—Assessing Compliance with Tobacco Advertising, Promotion and Sponsorship Bans in Poland

**DOI:** 10.3390/ijerph18041976

**Published:** 2021-02-18

**Authors:** Kinga Polanska, Dorota Kaleta

**Affiliations:** Department of Hygiene and Epidemiology, Medical University of Lodz, 90-752 Lodz, Poland; dorota.kaleta@umed.lodz.pl

**Keywords:** tobacco, e-cigarettes, point of sale advertising, compliance, public health, tobacco control

## Abstract

The objective of this study was to evaluate compliance with the ban on tobacco and e-cigarette products advertising at point of sale (POS) before and after amendment of the Polish Tobacco Control Act. Data were collected, using an observation checklist, between March and October 2014 (*n* = 1450 POS) and between March and October 2019 (*n* = 1320 POS). Ban on tobacco and e-cigarette advertising at POS is commonly violated in Poland. In all POS, at least one form (including tobacco products display) of tobacco advertising was found in 2014 and in 2019. The most common types of tobacco advertising in 2014 were change and counter mats (61%, 42%), posters (38%) and illuminated banners (37%). In 2019, a decrease in promoting tobacco products in the form of mats (*p* ≤ 0.001), posters and boards (*p* < 0.001) but an increase in video screens were observed (from 8% in 2014 to 30% in 2019; *p* < 0.001). A significant increase in the presence of any e-cigarette ads, including e-cigarette displays, illuminated banners and video screens, was observed in 2019 as compared to 2014 (90% vs. 30%; 89% vs. 20%; 31% vs. 2%; 31% vs. 0.5%; *p* < 0.001). The minimum age or a no-sale-to-minors signs for tobacco and e-cigarettes were not sufficiently placed in POS to comply with the Act. Poor enforcement of the ban on tobacco and e-cigarette ads at POS provides the tobacco industry with an opportunity to promote their products using unlawful ways. There is a need to educate the public, retailers and civil society with respect to their legal responsibilities and roles.

## 1. Introduction

The tobacco epidemic is one of the major threats to public health. It is responsible for nearly 8 million deaths a year worldwide [1]. Tobacco consumption, including direct and second-hand smoking, also continues to be the leading preventable cause of death in Poland, being responsible for an estimated 21% of all deaths [2]. Although smoking rates have decreased since 2000, around 31% of men and 20% of women were currents smokers in 2018 [3]. The data on tobacco smoking prevalence among children and young people are also highly alarming. Results of the 2017/2018 Health Behaviour in School-Aged Children (HBSC) survey indicate that 22% of 11–15 years old youth in Poland have already smoked, while 9% of them smoked within the last 30 days preceding the survey [4].

E-cigarettes have been available on the Polish market since early 2008 and they have gained unexpectedly large popularity, especially among young people. The cross-sectional study conducted in Poland among teenagers aged 15–19 indicated that e-cigarette trial and past 30-day exclusive e-cigarette use significantly increased over time (2010–2011: 2%; 2013–2014: 8%; 2015–2016: 11% (*p* < 0.05)). Dual use increased from 4% in 2010–2011 to 23% in 2013–2014 (*p* < 0.05) [5]. Another study conducted in Poland concluded that a high proportion of the youth is susceptible to e-cigarette use (68% of never and 78% of ever e-cigarette users) [6].

The World Health Organization Framework Convention on Tobacco Control (WHO FCTC) recommends implementation of comprehensive bans on tobacco advertising, promotion and sponsorships (TAPS) as part of an effective set of tobacco control policies [7,8]. Article 13 of the WHO FCTC and its Guidelines require a comprehensive ban on all TAPS, including point of sale (POS) tobacco product displays. In Poland, Article 8 of the Tobacco Control Act (TCA) in 1995 established a broad ban on tobacco advertising and promotion and was gradually expanded to include, in 1999 (with effect from 2001), all print media and billboards in addition to radio and television broadcasts [9]. The 1995 TCA forbids tobacco companies from advertising or promoting tobacco products and accessories, and the products that imitate tobacco products or accessories, or symbols alluding to the consumption of tobacco. The ban also forbids tobacco companies from promoting tobacco products or sponsor sporting, cultural, educational, health, social and political activities. According to the TCA, tobacco advertising is any public dissemination of images of tobacco product brands or logos thereof, and the names and logos of tobacco companies are substantially the same as the names and logos of tobacco products used for tobacco brands promotion. However, commercial information exchanged by the manufacturers, distributors and traders of tobacco products is not included in the definition of the advertising. Moreover, tobacco product promotion is defined as the public, free-of-charge distribution of tobacco products or accessories, orchestrating sampling and bonus sales of tobacco products, along with other schemes of public encouragement or consumption of tobacco products. The law also regulates obligatory placement of the minimum age or a no-sale-to-minors sign in POS. 

Until 2016, e-cigarettes were not subject to any legal regulations in Poland, which means they were easily available to minors (as there was no age limit for buying and using them) and that e-cigarette advertising, promotion and sponsorship was widespread all over the country [5,6]. Therefore, as there were no regulations and all forms of e-cigarette advertising, promotion and sponsorship were legal, e-cigarette companies focused on them and paid less attention to promotion of their products at POS. It was only the amendment of the TCA, which was announced on July 22, 2016, that referred all legal regulations to the same extent to both traditional cigarettes and e-cigarettes—thus, defined the minimum age at which one can buy cigarettes (18 years of age), banned advertising, promotion and sponsorships and stated that at all retail outlets, visible and legible information shall be placed that reads: “No sale of tobacco products, electronic cigarettes or refill containers to persons under 18” [10]. 

While compliance with the tobacco and e-cigarette advertising, promotion and sponsorship ban seems to be high in traditional advertising media such as broadcast, print and billboards, there are some legal and enforcement issues regarding retail tobacco products advertising. POS tobacco and e-cigarette product displays and distribution of the so called “commercial information” exchanged by the manufacturers, distributors and traders of tobacco products are allowed under the TCA. As a result, visual forms that popularise specified tobacco and e-cigarette products and encourage their purchase have been commonly found at POS for a long time. However, visual forms (posters, illuminated boxes) are illegal because they contain elements that encourage to purchase tobacco and e-cigarette products. 

Although tobacco control policies have been adopted worldwide, effective implementation continues to be a major challenge [11]. Policy implementation is understood as the stage that focuses on turning policy intentions into actions [12]. One of the most commonly pointed out barriers to tobacco control policy implementation is tobacco industry interference [11]. The conceptual framework proposed by Hoe et al., dedicated to low-and-middle income courtiers, but in our opinion also reasonable for developed ones, pointed out the following factors that have been shown to influence implementation fidelity: political commitment, institutional capacity and operational effectiveness, social climate and tobacco industry interference [11].

To our best knowledge only, a few assessment studies have been implemented to monitor TAPS (with no studies focusing on e-cigarettes) at POS worldwide, while no such studies at all have been launched in Poland [13,14,15,16,17,18,19,20].

Considering the above, the main objective of the study was to evaluate compliance with the ban on tobacco and e-cigarette advertisements at POS before and after amendment of the TCA. Several practices of tobacco and e-cigarette sales were also evaluated.

## 2. Material and Methods

### 2.1. Study Design and Procedure

This study was conducted at POS in Lodz (central Poland). To assess tobacco and e-cigarette advertising, promotion and sponsorship at POS, an observation checklist based on the TAPS compliance assessment guidance by the Johns Hopkins Bloomberg School of Public Health, the Campaign for Tobacco-Free Kids and the International Union against Tuberculosis and Lung Disease was implemented [21,22,23]. This is a widely used standardised observation instrument [13]. Collection tools, including an observation checklist, and procedures for conducting observation that described every step of the process were adapted considering provisions of the laws that regulate tobacco and e-cigarette advertising, promotion and sponsorship activities in Poland. In February 2014 and in February 2019, a five-hour training and fieldwork for 15 study data collectors who were technical assistants of the Medical University of Lodz, Poland, was completed. They were trained to recognise tobacco and e-cigarette advertising activities, to use an observation checklist and to document the relevant information. The training ended with an examination in which the data collectors visited five different stores and performed all the procedures according to the purpose of the study; the stores were revisited by the supervision team so as to verify correctness of the obtained information. 

Data were collected between March and October 2014 and between March and October 2019 (before and after amendment of the TCA that introduced regulations dedicated to e-cigarettes, announced on 22 July 2016) [9]. Each POS was visited by one member of the survey team; however, to guarantee reliability of the study, 5% of the stores were revisited by other collectors. Time spent per POS was approximately 10 to 15 min. The data collector observed tobacco and e-cigarette ads by walking through every aisle in the store, waiting in a line or making a small purchase. The owners of the shop were not informed in advance about the store visit to avoid any “preparation” (they might have altered their store displays if they had known the assessment was going to take place). Thanks to this, reliability and validity of the data collected were guaranteed [14,15].

Ethical considerations: observational studies do not require any ethical approvals, nor a prior informed consent form the vendors to complete the observation.

### 2.2. Study Area and Data Collection Process

Lodz is the fourth largest city of Poland with approximately 700 thousand inhabitants (706 thousand in 2014 and 685 thousand in 2019). Tobacco and e-cigarette products have been easily accessible in Poland. They have been sold in kiosks, general stores, groceries and delis, supermarkets, tobacco or liquor stores and gas stations. Information on the number and location of retail outlets offering tobacco products in 2014 was based on source data from the Lodz City Council, Regional Office of Statistics in Lodz register. Out of 7823 retail outlets registered in Lodz, 3649 sold tobacco products. Of these retail outlets, 1450 were selected according to the following procedure. As the first step, the available list of the retail outlets was verified, and the stores were grouped according to their type (kiosks, general stores, groceries and delis, supermarkets, tobacco or liquor stores and gas stations). In the next step, every third retail outlet from each group was selected for a visitation. Finally, from the remaining, not selected outlets, 10% were additionally selected to guarantee geographical representation (so the selected retail outlets, with information about the type of outlet, were marked on the map and if the representation of some type of retail outlet was not met in a specific location, additional outlets were selected). In 2014, e-cigarettes were sold in 403 of those visited outlets. For the repeated visits (in 2019) the same outlets were assigned; however, considering the fact that some of them were not accessible during the study (due to the following reasons: went out of business or changed their profile and as the consequence did not offer tobacco or e-cigarette products any longer), 1320 were visited (91%). All of the 1320 retail outlets sold tobacco products, and in 650 retail outlets, e-cigarettes were also available. 

### 2.3. Measures

We observed the presence of tobacco and e-cigarette ads at POS including pack displays in any form (yes/no), location of the advertisement including presence of exterior advert (on-building, yes/no) and its type: an illuminated banner, board, poster, sticker, video screen, change mat, counter mat or a product or an accessory that imitates tobacco or e-cigarettes, as well as other advertising materials like dangles, furniture, clocks, etc.

We also evaluated required-by-law presence of the minimum age or a no-sale-to-minors sign (yes/no) and tobacco or e-cigarette products displays in close proximity to, and in the same visual field with products for children, such as sweets or toys (yes/no).

### 2.4. Statistical Analysis 

The data were being entered into Excel data analysis software on a daily basis by the field investigators and were submitted to a supervisor. Once the data collection process had been completed, 15% of records were randomly checked to confirm that they were clearly recorded, complete and consistent across responses. Prevalence of tobacco and e-cigarette ads in POS (*n*, %; 95%CI) was assessed. The *p*-value for the difference between 2014 and 2019 was calculated. The standard significance level *p* < 0.05 was selected for the interpretation of the results. STATISTICA version 10.0 (Dell Software, Arizona, CA, USA) was used to perform the statistical analysis.

## 3. Results

Descriptive characteristics of POS visited during 2014 and 2019 are presented in Table 1. There was no significant difference in the proportion of retail outlet types between the two data collection points (*p* > 0.05). Grocery and deli stores (35% in 2014 and 33% in 2019) as well as kiosks and newsstands (23% in 2014 and 21% in 2019) were the most frequently visited ones. 

### 3.1. Tobacco Advertisements

In all POS, at least one form (including tobacco products display) of TAPS was found in 2014 and in 2019 (Table 2). Prevalence of exterior adverts on a building was low: 2.4% in 2014 and decreased to 1% in 2019 (*p* = 0.003). The most common types of TAPS violating legal provisions in 2014 included: change mats (61%), counter mats (42%), posters (38%), various types of illuminated banners (37%) and boards (33%). In 2019, a decrease in promoting tobacco products in a form of mats (*p* ≤ 0.001), posters and boards (*p* < 0.001) but an increase in video screens were observed (from 8% in 2014 to 30% in 2019; *p* < 0.001). The products or accessories that imitate tobacco products were a much less popular type of TAPS in 2019 as compared to 2014 (2% vs. 10%; *p* < 0.001). 

### 3.2. E-Cigarette Advertisements

In 2014, of 1450 retail outlets selling tobacco cigarettes, 403 (28%) also sold e-cigarettes, whereas in 2019, almost 50% shops sold both products (*p* < 0.001). A significant increase in the presence of any e-cigarette ads, including e-cigarette displays, illuminated banners and video screens, was observed in 2019 as compared to 2014 (90% vs. 30%; 89% vs. 20%; 31% vs. 2%; 31% vs. 0.5%; *p* < 0.001). E-cigarettes were also promoted in 2019 in a form of change and counter mats, which were not used in 2014 (32% vs. 0%; *p* < 0.001). 

### 3.3. Sales Practices

The minimum age or a no-sale-to-minors signs for tobacco cigarettes were found in 50% of POS visited in 2014 and in 60% of POS visited in 2019 (*p* < 0.001) (Table 3). The same information but dedicated also to e-cigarettes was not found in 2014 (as this was not regulated by law at that time) and in 29% of POS checked in 2019 (regulated by law since 2016) (*p* < 0.001). Tobacco products were placed in close proximity to products for children in 23% of the outlets visited during the first and in 18% of the outlets visited during the second wave of the study (*p* < 0.001). The opposite pattern was observed for e-cigarette placement (16% in 2014 vs. 27% in 2019; *p* < 0.001). 

## 4. Discussion

The current study reveals that despite legislation, tobacco and e-cigarette ads still exist in POS in Poland. The industries mostly promote their products in a form of illuminated banners and video screens. Also, change and counter mats promoting tobacco and e-cigarettes are still very popular. The minimum age or a no-sale-to-minors sign for tobacco and e-cigarettes was not sufficiently placed in POS so as to comply with the Act. 

This research demonstrates that not much improvement in enforcement of the law (despite amendment of the Act in 2016) has been achieved over the years. According to the extensive research, the best practice would be a complete ban on tobacco and e-cigarette advertising, promotion and sponsorship [24]. In some places around the world, this is a reality. However, the use of retail store advertising to promote tobacco and e-cigarette products is also observed in other studies [13]. It is crucial to fully implement recommendations of the FCTC and eliminate possible loopholes or adapt regulations to the emerging market for new products or forms of ads. Since the ban on tobacco or e-cigarette advertising became effective, the obvious examples of advertising (through TV, press, posters) have disappeared. In addition, high fines have solved the problem. But now, it is POS tobacco and e-cigarette products displays that are extensively used by the industry to advertise their products. 

The tobacco and e-cigarette industries use extraordinarily smart and aggressive marketing tactics to attract new consumers, but also to retain those who are already smokers [25,26,27,28,29,30]. Ads or so-called “corporate social responsibility” programmes sponsored by tobacco or e-cigarette corporations have been proven to be carefully designed to increase social acceptability of the products, increase the amount of consumption among current smokers or users, reduce willingness to quit and increase the risk of relapse among quitters [11,31,32,33]. Existing studies have revealed strong associations between exposure to advertising, including POS product displays, with initiation, susceptibility to and progression of smoking or e-cigarette use especially among youths [13,26,27,28,29,30].

Based on the observations of the assessment team, POS is currently exploited for tobacco and e-cigarette advertising and promotion in Poland, because other channels of its promotion have been blocked. Types of advertising have been developed so as to pretend to be “information on tobacco or e-cigarette products at POS”, which is less conspicuous. On the other hand, matts, coin trays, illuminated boards and video displays contain elements of promotion to attract consumer attention and promote tobacco and e-cigarette brands or products. They are colourful, much larger than required and all just to show the product to be sold. In addition to the information about the product, they contain words that may be deemed advertising, and messages longer than the names of the products. These elements popularise tobacco and e-cigarette products, and some even encourage people to buy them. Additionally, kiosks are a special case, as many of them are made of transparent materials and the contents are clearly visible from the street and are extensively used by the industry to advertise their products and help manufacturers and retailers use their marketing strategies that circumvent the ban of TAPS. 

The major finding of the study is the increase in various forms of e-cigarette advertising at POS. We would like to underline that before 2016, there were no legal regulations dedicated to e-cigarettes in Poland so e-cigarette companies focused on the range of available types of advertising, promotion and sponsorship (especially the billboards and media advertising were very popular). After amendment of the TCA, e-cigarettes have been under the same regulations as traditional cigarettes with a ban on advertising, promotion and sponsorship. Therefore, they shifted to advertising at POS, taking advantage of the gap in the law saying that commercial information exchanged by the manufacturers, distributors and traders of tobacco products is not included in the definition of advertising. Moreover, considering the unexpected popularity of e-cigarettes, especially among the youth, they focused on these products even more to obtain even more customers [4]. Placing e-cigarettes near the products for children, which was observed in our study, is quite an appalling practice. However, it is rather related to the general layout of products in a store—usually these two types of products are placed near the till. 

It is necessary to discipline producers (the owners of these ads) and vendors who misuse the law by transforming information about products into advertisements. The right and obligation to monitor advertisements at POS rests with Trade Inspection, or more generally, with the Office of Competition and Consumer Protection (UOKIK). Every other public service: the city guard, the police and the State Sanitary Inspection, is entitled to intervene (for the sake of preventing violation of law in a public place). Prosecution of those advertising forms often fails as a result of hesitation of the involved organisations. A vicious circle occurs: ineffective prosecution causes advertisers to become less apprehensive, while more widespread advertising obtains a semblance of legality and advertisers become even bolder and the authorities more apprehensive. The wording of the TCA may not be specific enough, but its intention is clear: legislators intended that there should be a total ban on all forms of tobacco and e-cigarette promotion. Therefore, the strategies described above are illegal, but continue to be unpunished due to the enforcement problems. 

The framework developed by Hoe et al. outlines four interacting components and factors that have been shown to contribute to increase implementation fidelity [11]. The first proposed component relates to the political commitment from the high-level decision makers in the country. As an example, favourable changes in the government can provide leaders who are more keen to strengthen the existing law and establish more effective law enforcement methods. The second component is dedicated to the institutional capacity and operational effectiveness of the country, state/province or municipality. Effective implementation requires networks that can provide a critical platform for information exchange and sharing resources, knowledge and expertise. The presence of an implementation plan that clearly defines roles and responsibilities as well as sufficient investments to ensure adequate resources and workforce is also crucial. The third component, which is the social climate, is characterised by norms, practices and beliefs throughout the society that increase the likelihood of policy compliance. The presence of advocacy groups may increase public awareness, sensitise decision makers and effectively monitor for non-compliance. Finally, the fourth component refers to tobacco industry interference. Tobacco or e-cigarettes companies frequently employ an array of tactics including lobbying, political campaign contributions, corporate social responsibility activities and litigation to influence the policy-making process. As it was pointed out by Hoe et al., these four components inter-relate [11]. 

When the amendment of the TCA was announced in Poland, most resources, including the public campaigns, were focused on information and education of the society and the owners of retail outlets on the age limit dedicated to e-cigarettes but less on different forms of advertising that may show up that may not be compliant with the law in force. This should be the target action to be taken to eliminate illegal advertising and promotion at POS effectively. Of all the tobacco retail outlets, only 50% had “a no sales to minors” signs visible in 2014, and although some improvement with that respect was observed in 2019 (60% outlets placed that sign), it is still associated with poor enforcement of the law. The worst situation is observed in the case of e-cigarettes. Despite the legal regulation, that has existed since 2016 and states that at the retail outlets, visible and legible information shall be placed that reads: “No sale of tobacco products, electronic cigarettes or refill containers to persons under 18,” such signs were found only in one third of the visited places. When regulations are clear, monitoring is regular and penalties are severe, the law is obeyed. On the other hand, the lack or incomprehensive provisions of the ban on TAPS at POS leads to poor enforcement, giving the tobacco and e-cigarette industries an opportunity to exploit the situation and promote their products using various deceitful ways.

Our study has several strengths. Firstly, according to our knowledge, this is the first study in Poland that has evaluated effectiveness of existing legislation on the ban on tobacco and e-cigarette advertising at POS. Secondly, repeated observations allowed us to capture changes in the intensity of marketing and frequency of violations of the law in POS over time. Thirdly, the checklist and protocol are based on valid tools developed by experts in the field [21]. Our study considered audit methods for assessing tobacco and e-cigarette marketing and products in the POS [34]. Moreover, visiting the stores without notice provided an opportunity to collect data “in a real situation” not influenced by store outlets owners. Finally, the data used in the current analysis are based on the local register of POS, which covers a sufficient number of POS from the entire area of Lodz, assuring generalizability of results for urban areas. However, its applicability to rural areas may be limited.

One of the limitations of our study is that some marketing strategies, like providing free samples or offering gifts or bulk sales of cigarettes, could not be recorded during the single short observation and need a different approach and further research. We were also unable to consider in the checklist and disclose all industry tactics that were difficult to categorise, like for instance: using price stickers to hide health warnings. However, from a public health perspective, our data may constitute a sufficient reason to take legal action.

## 5. Conclusions and Recommendations for Further Actions

Results of the current study are alarming. A huge number of illegal adverts and various types of marketing activities in POS require more targeted interventions. Legislative priorities include amending the act, while practice priorities comprise improved activities of relevant institutions, including the UOKIK, police, city guard and launching an extensive education campaign. First of all, enforcement of existing legislation must be improved by clearly defining roles and responsibilities of selected agencies for monitoring and enforcement. The government must ensure that those who are responsible for monitoring and enforcing legislation are appropriately trained to have a full understanding of the law and its implications. They should be aware of the tactics of the tobacco/e-cigarette industry and its allies trying to circumvent the legislation. There is a need to educate the public, retailers, civil society and stakeholders about the existence of the tobacco and e-cigarette advertising, promotion and sponsorship restrictions and the status of compliance as well as their legal responsibilities and roles. Civil society can assist governmental agencies in monitoring breaches of tobacco control legislation. This has been an important element of restrictions enforcement in other countries. This requires making information available to the general public and enlisting the help and cooperation of relevant non-governmental organisations (NGOs). NGOs can be very effective partners, but they may need some support to be able to offer the most effective assistance. Furthermore, to play their part, the general public must know how to report breaches and to whom. A conducive social climate may encourage politicians to be more committed and lessen the amount of resources required to enforce the policy and fuel support to prevent tobacco industry interference. Moreover, loopholes or deficiencies in the law must be eliminated. There is an urgent need to enact tobacco and e-cigarette advertising, promotion and sponsorship bans that are strict and comprehensive. Some problems of enforcement may be due to the unclear definitions of advertising, sponsorship and promotion in the Act itself. The most effective course of action would be to use the FCTC definitions that are based on the best international evidence and practice to minimise loopholes.

## Figures and Tables

**Table 1:** Descriptive characteristic of points of sale.

Retail Store Type	March–October 2014 N = 1450	March–October 2019 N = 1320	*p*-Value
	*n* (%) (95% CI)		
Kiosk/newsstand	337 (23.2%) (21.0–25.4)	277 (21.0%) (18.8–23.2)	0.15
Gas station	75 (5.2%) (4.1–6.3)	84 (6.4%) (5.1–7.7)	0.18
Grocery store and deli	504 (34.8%) (32.4–37.3)	435 (33.0%) (30.5–35.5)	0.32
Tobacco or liquor store only	108 (7.4%) (6.1–8.8)	92 (7.0%) (5.6–8.4)	0.63
General store	201 (13.9%) (12.1–15.7)	198 (15.0%) (13.1–16.9)	0.39
Supermarket	205 (14.1%) (12.3–15.9)	219 (16.6%) (14.6–18.6)	0.07
Other	20 (1.4%) (0.8–2.0)	15 (1.1%) (0.5–1.7)	0.57

**Table 2:** Prevalence of tobacco and e-cigarettes advertisements.

Types of Tobacco/E-Cigarette Advertisements	March–October 2014	March–October 2019	*p*-Value
	*n* (%) (95% CI)		
**Tobacco Advertisements**	**N = 1450**	**N = 1320**	
Presence of any tobacco ads	1450 (100.0%) (100.0–100.0)	1320 (100.0%) (100.0–100.0)	No difference
Power wall, tobacco products displays	1450 (100.0%) (100.0–100.0)	1320 (100.0%) (100.0–100.0)	No difference
External ads	36 (2.5%) (1.7–3.3)	13 (1.0%) (0.5–1.5)	0.003
Illuminated Banners	532 (36.7%) (35.5–37.9)	436 (33.0%) (30.5–35.5)	0.04
Boards	479 (33.0%) (30.6–35.4)	224 (17.0%) (15.0–19.0)	<0.001
Posters	551 (38.0%) (35.5–40.5)	205 (15.5%) (13.5–17.5)	<0.001
Stickers	173 (11.9%) (10.0–13.8)	187 (14.2%) (13.6–17.5)	0.08
Video screens	109 (7.5%) (6.1–8.9)	400 (30.3%) (27.8–32.8)	<0.001
Change mats	890 (61.4%) (59.6–63.2)	749 (56.7%) (54.0–59.4)	0.01
Counter mats	606 (41.8%) (39.3–44.3)	395 (29.9%) (27.4–32.4)	<0.001
Products or accessories that imitate tobacco products *	139 (9.6%) (8.1–11.1)	25 (1.9%) (1.2–2.6)	<0.001
Other	91 (6.3%) (5.1–7.6)	25 (1.9%) (1.2–2.6)	<0.001
**E-Cigarette Advertisements**	**N = 403**	**N = 650**	
Presence of any e-cigarette ads	121 (30.0%) (25.5–34.5)	598 (92.0%) (89.9–94.1)	<0.001
Power wall, e-cigarette displays	80 (19.9%) (16.0–23.8)	580 (89.2%) (86.8–91.6)	<0.001
External ads	6 (1.5%) (0.3–2.7)	6 (0.9%) (0.2–1.6)	0.40
Illuminated Banners	8 (2.0%) (0.6–3.4)	201 (30.9%) (27.4–34.5)	<0.001
Boards	10 (2.5%) (1.1–3.9)	30 (4.6%) (3.0–6.2)	0.08
Posters	10 (2.5%) (1.8–3.2)	32 (4.9%) (3.2–6.6)	0.05
Stickers	8 (2.0%) (0.6–3.4)	8 (1.2%) (0.4–2.0)	0.33
Video screens	2 (0.5%) (0.0–1.2)	201 (30.9%) (27.4–34.5)	<0.001
Change mats	0 (0.0%) (0.0–0.0)	205 (31.5%) (27.9–35.1)	<0.001
Counter mats	0 (0.0%) (0.0–0.0)	205 (31.5%) (27.9–35.1)	<0.001
Products or accessories that imitate e-cigarettes	5 (1.2%) (0.1–2.3)	15 (2.3%) (1.2–3.4)	0.23
Other	0 (0.0%) (0.0–0.0)	4 (0.6%) (0.0–1.2)	0.11

* Imitating a pack of cigarettes or e-cigarettes, a pen or a pencil.

**Table 3:** Sales practices.

Types of Sales Practices	March–October 2014	March–October 2019	*p*-Value
	*n* (%) (95% CI)		
Tobacco Cigarettes	N = 1450	N = 1320	
Minimum age or no sale to minors sign is present	731 (50.4%) (47.8–53.0)	792 (60.0%) (57.4–62.6)	<0.001
Placed near to products for children	339 (23.4%) (21.2–25.6)	238 (18.0%) (15.9–20.1)	<0.001
**E-Cigarettes**	**N = 403**	**N = 650**	
Minimum age or no sale to minors sign is present	0 (0.0%) (0.0–0.0)	190 (29.2%) (25.7–32.7)	<0.001
Placed near to products for children	65 (16.1%) (12.5–19.7)	172 (26.5%) (23.1–29.9)	<0.001

## Data Availability

The data presented in this study are available on request from the corresponding author.
